# Genetic Mapping and Functional Studies of a Natural Inhibitor of the Insulin Receptor Tyrosine Kinase: The Mouse Ortholog of Human α_2_-HS Glycoprotein

**DOI:** 10.1155/EDR.2000.249

**Published:** 2000

**Authors:** Vivian J. Cintrón, Minoru S. H. Ko, Kenneth D. Chi, Jason P. Gross, Pothur R. Srinivas, Anton Scott Goustin, George Grunberger

**Affiliations:** 1 Department of Biological Sciences Wayne State University School of Medicine Detroit MI 48201 USA; 2 Center for Molecular Medicine and Genetics Wayne State University School of Medicine Detroit MI 48201 USA; 3 Department of Internal Medicine Wayne State University School of Medicine Detroit MI 48201 USA

## Abstract

Fetuin/α_2_-HS glycoprotein (α_2_-HSG) homologs have been identified in several species including rat, sheep, pig, rabbit, guinea pig, cattle, mouse and human. Multiple physiological roles for these homologs have been suggested, including ability to bind to hydroxyapatite crystals and to specifically inhibit the tyrosine kinase (TK) activity of the insulin receptor (IR). In this study we report the identification, cloning, and characterization of the mouse Ahsg gene and its function as an IR-TK inhibitor. Genomic clones derived from a mouse Svj 129 genomic library were sequenced in order to characterize the intron–exon organization of the mouse Ahsg gene, including an 875 bp subclone containing 154 bp upstream from the transcription start site, the first exon, and part of the first intron. A second genomic subclone harboring a 3.45 kb Bgl II fragment contained exons 2, 3 and 4 in addition to two adjacent elements within the first intron-a repetitive element of the B1 family (92 bp) and a 271 bp tract of (T,C)*n* * (A,G)*n*. We have mapped mouse Ahsg at 16 cM adjacent to the Diacylglycerol kinase 3 (Dagk3) gene on chromosome 16 by genotyping interspecific backcross panels between C57BL/6J and *Mus spretus*. The position is syntenic with human chromosome 3q27, where the human AHSG gene resides. Using recombinant mouse α_2_-HSG expressed from a recombinant baculovirus, we demonstrate that mouse α_2_-HSG inhibits insulin–stimulated IR autophosphorylation and IR-TKA *in vitro*. In addition, mouse α_2_-HSG (25μg/ml) completely abolishes insulin-induced DNA synthesis in H-35 rat hepatoma cells. Based on the sequence data and functional analysis, we conclude that the mouse Ahsg gene is the true ortholog of the human AHSG gene.


*Int. Jnl. Experimental Diab. Res.*, Vol. 1, pp. 249–263
Reprints available directly from the publisher
Photocopying permitted by license only

© 2001 OPA (Overseas Publishers Association) N.V.
Published by license under
the Harwood Academic Publishers imprint,
part of The Gordon and Breach Publishing Group.
Printed in Malaysia.

### Genetic Mapping and Functional Studies of a Natural Inhibitor of the Insulin Receptor Tyrosine Kinase: The Mouse Ortholog of Human $\alpha_2$-HS Glycoprotein

VIVIAN J. CINTRÓN[a], MINORU S. H. KO[b], KENNETH D. CHI[b], JASON P. GROSS[b], POTHUR R. SRINIVAS[c], ANTON SCOTT GOUSTIN[b] and GEORGE GRUNBERGER[b,c,*]

[a]*Department of Biological Sciences,* [b]*Center for Molecular Medicine and Genetics,* [c]*Department of Internal Medicine, Wayne State University School of Medicine, Detroit, MI 48201, USA*

*(Received in final form 19 May 2000)*

**Fetuin/$\alpha_2$-HS glycoprotein ($\alpha_2$-HSG) homologs have been identified in several species including rat, sheep, pig, rabbit, guinea pig, cattle, mouse and human. Multiple physiological roles for these homologs have been suggested, including ability to bind to hydroxyapatite crystals and to specifically inhibit the tyrosine kinase (TK) activity of the insulin receptor (IR). In this study we report the identification, cloning, and characterization of the mouse Ahsg gene and its function as an IR-TK inhibitor. Genomic clones derived from a mouse Svj 129 genomic library were sequenced in order to characterize the intron–exon organization of the mouse Ahsg gene, including an 875 bp subclone containing 154 bp upstream from the transcription start site, the first exon, and part of the first intron. A second genomic subclone harboring a 3.45 kb Bgl II fragment contained exons 2, 3 and 4 in addition to two adjacent elements within the first intron-a repetitive element of the B1 family (92 bp) and a 271 bp tract of (T,C)*n* * (A,G)*n*. We have mapped mouse Ahsg at 16 cM adjacent to the Diacylglycerol kinase 3 (Dagk3) gene on chromosome 16 by genotyping interspecific backcross panels between C57BL/6J and *Mus spretus*. The position is syntenic with human chromosome 3q27, where the human AHSG gene resides. Using recombinant mouse $\alpha_2$-HSG expressed from a recombinant baculovirus, we demonstrate that mouse $\alpha_2$-HSG inhibits insulin-stimulated IR autophosphorylation and IR-TKA *in vitro*. In addition, mouse $\alpha_2$-HSG (25 μg/ml) completely abolishes insulin-induced DNA synthesis in H-35 rat hepatoma cells. Based on the sequence data and functional analysis, we conclude that the mouse Ahsg gene is the true ortholog of the human AHSG gene.**

*Keywords*: $\alpha_2$-HS glycoprotein; Fetuin; Mapping; Insulin receptor; Tyrosine kinase inhibitor

*Data Base Accession Numbers*: AF025820, AF025821

### INTRODUCTION

Heremans and Schmid described $\alpha_2$-HSG for the first time in 1960,[1,2] as a 49–60 kD human plasma glycoprotein secreted into the circulation by the liver at a concentration of 0.4–0.85 g/L. Human $\alpha_2$-HSG is a negative acute phase

*Address for correspondence: Center for Molecular Medicine and Genetics, Wayne State University, School of Medicine, 3216 Scott Hall, 540 E. Canfield Ave., Detroit, MI 48201, USA. Tel.: 313-993-7385, Fax: 313-993-6839, e-mail: g.grunberger@wayne.edu reactant protein[3,4] and altered concentrations of $\alpha_2$-HSG have been reported in several disease conditions including Paget's disease, osteogenesis imperfecta, lymphoma, leukemia and myelofibrosis.[5,6] Several functions have been attributed to $\alpha_2$-HSG, including its involvement in immune response,[7] the chemotactic response of macrophages,[8,9] enhancement of phagocytic function of human monocytes,[10,11] bone mineralization,[12,13] bone accumulation,[14,15] calcification[16,17] and as an inhibitor of insulin receptor tyrosine kinase activity [IR-TKA; [18]]. Recently, Jahnen-Dechent *et al.*[19] demonstrated that mice deficient for $\alpha_2$-HSG can develop ectopic microcalcifications in soft tissues, supporting the idea that $\alpha_2$-HSG may operate as an inhibitor of apatite crystal growth *in vivo*.

Classified as a member of the cystatin superfamily,[20] human $\alpha_2$-HSG is synthesized as a type I secreted glycoprotein with a signal sequence of 18 amino acids[21] and three major domains – two *N*-terminally located cystatin domains, D1 and D2 (116–118 amino acids), and a single, proline-rich domain D3[106–115 residues; [20,22–24]]. Many key amino acid residues and the position of cysteine residues are perfectly conserved between human $\alpha_2$-HSG and the fetuins of bovine, sheep, pig, rat and mouse origin,[25,20,26,21,27–29] suggesting that the fetuins may be the homologs of human $\alpha_2$-HSG.

Rat fetuin, originally named pp63,[30,31] secreted by rat hepatocytes in the phosphorylated state,[30] inhibits insulin-stimulated IR-TKA.[32] The gene for rat fetuin maps to chromosome 11 and spans approximately 8 kb, containing seven exons separated by six introns of different sizes. The rat fetuin cDNA sequence is similar to the cDNA sequences of both human $\alpha_2$-HSG and bovine fetuin.[33]

When Yang *et al.*[34] first reported the deduced amino acid of the mouse fetuin cDNA, the question arose whether mouse fetuin was the true ortholog of human $\alpha_2$-HSG. They suggested that the mouse protein take the name $\alpha_2$-HSG instead of fetuin, because, unlike bovine fetuin, the mouse protein is not a major component synthesized by fetal liver.[34,35] Alignment of human and mouse $\alpha_2$-HSG reveals a 60% amino acid identity between the two proteins, with the majority of the identical residues found in the *N*-terminal two-thirds of the protein. Three *N*-linked glycosylation sites are present in mouse while only two are present in the human protein. Moreover, mouse $\alpha_2$-HSG is 22 residues shorter than human $\alpha_2$-HSG.[34]

In this study, we report the mouse Ahsg genomic structure derived from sequencing and restriction mapping of exons 1, 2, 3 and 4 contained in a contiguous 4.3 kb segment of the gene. We have also sequenced a 154 bp region upstream from the transcriptional start site. The chromosomal location of mouse Ahsg has been mapped to the proximal region of chromosome 16 at 16 centimorgans, adjacent to the gene Dagk3. Further, we demonstrate that recombinant mouse $\alpha_2$-HSG inhibits insulin-stimulated IR autophosphorylation, IR-TKA and DNA synthesis, confirming that mouse $\alpha_2$-HSG can play a role similar to the human homolog in modulating insulin action. Based on the structural features shared between the mouse and human genes, their syntenic chromosomal localization, and the IR-TK inhibitory activities shared between the two proteins, we suggest that the mouse Ahsg is not simply a family member, but the true ortholog of the human AHSG gene.

### MATERIALS AND METHODS

### Cloning of Mouse Ahsg cDNA into a Recombinant Baculovirus

A full-length cDNA encoding the entire 1035 nt open reading frame (ORF) was cloned from liver poly(A+) RNA using reverse transcriptase (RT)-polymerase chain reaction (PCR). The cDNA was amplified in two segments, a 533

bp *N*-terminal segment (using primers SSF1, 5′-GGATCCTGACATTTGCCCATTTTCC-3′OH, and SSB3, 5′-CGGTGTGGACCACGTTGGTATC-3′OH) and a 1095 bp *C*-terminal segment (using primers VIV1, 5′-CTGCCAATCCGCTCCACAAGG-TA-3′OH and VIV2, 5′-TGTGGTATTGCTTTGT-CAGTGGA-3′OH). A continuous cDNA of 1182 bp was then amplified from the two segments using PCR and short overlap extension (primers SSF1 + VIV2) and the PCR product cloned into plasmid pCR (Invitrogen, Carlsbad, CA); the plasmid was sequenced from a combination of dye primer and dye-terminator automated sequencing reactions. The Ahsg ORF was shuttled as a 1.23 kb BamHI-XbaI fragment into the baculovirus transfer vector pBlueBacIII (Invitrogen, Carlsbad, CA) after blunting both the XbaI end of the Ahsg ORF and the Hind3 site of pBlueBacIII with the Klenow fragment of *E. coli* DNA polymerase I (Pharmacia, Piscataway, NJ); resulting in a transfer vector, pMusBac$\alpha$3 (11,440 bp) in which the XbaI site is restored. This transfer vector (2 µg) was transfected into Sf9 cells (Gibco Life Technologies, Gaithersburg, MD) in the presence of 5 µg of the wild-type baculoviral DNA, and blue plaques selected from 2% agarose plugs stained in Bluo-Gal (Gibco Life Technologies, Gaithersburg, MD). Twice-purified viral plaques were expanded at 27°C, and recombinant viral stocks checked for homogeneity using PCR (baculoviral primers −44, 5′-TTTACTGTTTTCGTAACAGTTTTG-3′OH; and +778, 5′-CAACAACGCACAGAATCTAGC-3′OH).

Expanded recombinant baculoviral stocks were used to transfect cabbage looper High-Five™ cells (Invitrogen, Carlsbad, CA). Supernatants collected after 72 h were purified using affinity chromatography on jacalin lectin columns (Sigma, St. Louis, MO), washed with 100 mM Tris-pH 7.4 and eluted with 25 µM melibiose (Sigma, St. Louis, MO). Proteins separated on 12% SDS-PAGE were electroblotted to nitrocellulose and probed with a polyclonal rabbit antibody specific for rat fetuin.

### Screening of Genomic Clones Harboring the Mouse Ahsg Gene

An Svj 129 library, constructed in λDASH2 (Stratagene, LaJolla, CA) using spleen genomic DNA from male Svj mice, was screened for the mouse Ahsg gene (kind gift of Dr. Roger Askew, University of Cincinnati, OH). After infection of PLK-17 cells, plaques were generated at 50,000 plaques per plate, and lifted onto 137 mm Ø Nytran filters (0.45 µ, Schleicher and Schüll, Keene, NH). Filters were hybridized at high stringency (50% formamide, 43°C) with a [$^{32}$P]-labeled cDNA probe (1.2 kb) derived representing the mouse Ahsg cDNA.

### Chromosomal Mapping of Mouse Ahsg

Using the 3′-UTR of the mouse Ahsg cDNA as a target, a primer pair [sense 5′-CTTCAAAATC-TAGGCTTGATTCGG-3′OH and antisense 5′-GCTTTATGCCTTTCATCAAATTTGACCATT-3′ OH] was selected using the Primer Detective program.[36] Mouse genomic DNA (25 ng) was amplified using the above primers in a Thermal Cycler Model 9600 (Perkin-Elmer-Cetus, USA). The samples were heated at 95°C for 1 min and amplified by 35 cycles of denaturation (94°C for 30 sec), annealing (at the optimized temperature of 58°C for 30 sec), and extension (at 70°C for 1 min), followed by 3 min of extension (70°C) after the last cycle.[37] The Jackson Laboratory BSS interspecific backcross mouse panels were used to determine the mouse Ahsg chromosomal location.[38] A customized polyacrylamide gel electrophoresis apparatus (Nihin Eido, Japan) was used to obtained high resolution of the mapping results. A 28-well comb was specially designed to accommodate two interdigitated sample loadings with a 12-channel micropipetter. A total of 24 samples were loaded per 10% gel. The gels were run at 250 volts for 1 h, stained with 0.5 µg/ml of ethidium bromide and photographed by UV transillumination. Allele types, C57BL/6J or *M. spretus*, were scored by visual inspection of the gels and analyzed with the computer program, Map Manager.[39] The localization of the markers were determined according to the composite map of backcross panels.[38] The composite map of the BSS panel data contains 451 markers, including 49 MIT markers. In these composite maps, the average centimorgan length of the 95% confidence interval for these markers is 7.6 cM (BSS).

### Functional Studies of Recombinant Mouse $\alpha_2$-HSG with Respect to the Insulin Receptor

Insulin receptors were partially purified from the H-35 rat hepatoma cell line (ATCC, Rockville, MD) as described earlier.[40] Autophosphorylation of crude insulin receptors was assessed by preincubating various concentrations of $\alpha_2$-HSG in the presence of insulin (100 nM) for 30 min at 20°C. The phosphorylation reaction was initiated in the presence of [$^{32}$ P]-ATP (3000 Ci/mmol), $MnCl_2$ (8 mM), ATP (10 μM), PNPP (10 mM), and Na-orthovanadate (100 μM). Reactions were stopped after 10 min by boiling in the presence of 3% SDS and 100 mM DTT. Proteins were separated by SDS-PAGE 10% gel and the $^{32}$P incorporated was detected by autoradiography of the dried gel. An exogenous substrate, poly (Glu$^{80}$Tyr$^{20}$), was used to assess the IR-TK activity.

Insulin-induced DNA synthesis was monitored by incorporation of [$^{3}$H]-thymidine into H-35 cells. The cells were grown to 30% confluence and incubated in serum-free DMEM, containing 0.1% insulin-free BSA for 36 h and reincubated for 14 h with 100 nM insulin, in the presence of various concentrations of mouse $\alpha_2$-HSG. Cells were pulsed with 1 μCi/ml of [$^{3}$H]-thymidine (NEN-Dupont, Wilmington, DE) for 1 h, and then washed twice with ice-cold PBS. The cells were solubilized and the DNA precipitated with ice-cold TCA. The precipitates were collected on glass filters and washed twice with ice-cold 5% TCA. The radioactivity incorporated was quantitated in a scintillation counter.

### RESULTS

### Isolation of Genomic Clones Harboring the Mouse Ahsg Gene

A screening of 350,000 clones of the λDASH2 Svj 129 genomic library resulted in 11 independent clones. The relatedness of these clones to the mouse Ahsg gene was confirmed by PCR amplification of crude phage DNA using mouse Ahsg primers KOAF1 (5′-GCCCTTCGGAGTGGTG-TATGAGATG-3′OH) and KOAB10 (5′-ACGTTGG-TATCGTTGAACGGAGTC-3′OH) designed from targets in the cDNA sequence. PCR amplification of the clones resulted in a fragment of 0.95 kb which could be cleaved into three pieces using BstEII digestion. One clone (λKOA-1B) was selected for further characterization, and 10 μg of phage DNA prepared from plate lysates for restriction enzyme analysis. Restriction analysis performed on this clone using digestion with EcoRI or Bgl II (Fig. 1) suggested an insert size of 18.6–23.0 kb. Bgl II fragments of 6.0, 5.65, 3.42, 2.62 and 0.9 kb representing the mouse genomic insert were found (in addition to the λ arms); likewise, digest of λKOA-1B revealed EcoRI fragments of 6.6, 6.0, 4.85, 2.45, 1.7 and 1.42 kb (in addition to the λ arms). Two Bgl II fragments excised from an agarose gel (3.45 and 0.9 kb) were chosen for subcloning into the BamHI site of pGEM4Z, resulting in clones D and delta; both subclones were completely sequenced.

Sequence analysis of these two clones reveals that the smaller clone (delta) harbors the first exon (290 nt) in addition to 5′-regulatory elements up to the-154 position, as well as 431 nt of intron downstream of the first exon (Figs. 2A and 4A). We suggest that position 155 in Figure 2A be taken as the transcriptional start site (cap site) based on the alignment of

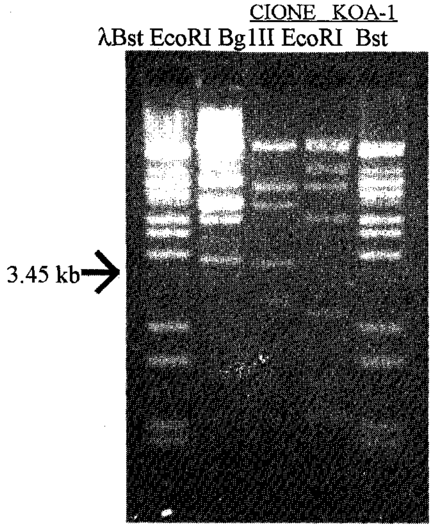

FIGURE 1 *Restriction analysis of Ahsg genomic clone λKOA-1B*. A 1.2 kb mouse Ahsg cDNA was used as a probe to screen a mouse genomic library. One of the eleven positive clones (λKOA-1B) was plaque-purified to homogeneity and 10 μg of DNA purified from plate lysates. DNA was cut with EcoRI or Bgl II and the DNA fragments separated on a 1% agarose gel. DNA in lanes 1 and 4 is a λBstE2 marker, lane 2 is λKOA-1B cut with Bgl II and lane 3 is λKOA-1B cut with EcoRI. Digestion with Bgl II generated bands ranging from 900 bp to about 8.5 kb. Two Bgl II fragments were selected for subcloning, a 3.45 kb Bgl II fragment (indicated by the arrow) and a 0.9 kb Bgl II band (not shown). Summation of the sizes of the non-vector fragments in lanes 2 and 3 imply that clone λKOA-1B, harbors a total of 18.6–23.0 kb of mouse DNA, more than twice the size of the known rat and human AHSG genes (7–8 kb; [49, 50]).

**A**

```

  1 agatctgttg gggagagatg atgtcctaac ttatttgctt ttccagagct gctqtttgca

                                  HNF-3β

 61 aggattattt ggaaccagaa cagaaatcgt cccacgcctt tacqcaattc cttcggcggg

                                              c/EBP-α

121 ctctgtcaga taaattaggc cctctgcccc tctattggtc tagctctcca agctgattat

              TATA box                     |----------- mRNA --------->

                                          +1

181 ccgggctgct cctgacattt gcccattttc cagggcctct ctggagcaac cATGAAGTCC

                     Primer SSF1 --->                      MetLysSer

241 CTGGTCTTGC TCCTTTGTTT TGCTCAGCTC TGGGGCTGCC AATCCGCTCC ACAAGGTACA

    LeuValLeuL euLeuCysPh eAlaGlnLeu TrpGlyCysG lnSerAlaPr oGlnGlyThr

301 GGACTGGGTT TTAGAGAATT GGCTTGTGAT GATCCAGAAG CGAAGCAAGT AGCTTTGTTG

    GlyLeuGlyP heArgGluLe uAlaCysAsp AspProGluA laLysGlnVa lAlaLeuLeu

361 GCCGTGGACT ACCTCAATAA TCATCTTCTT CAGGGATTCA AACAGGTCTT GAATCAGATC

    AlaValAspT yrLeuAsnAs nHisLeuLeu GlnGlyPheL ysGlnValLe uAsnGlnIle

421 GACAAAGTCA AGGTGTGGTC TCGGgtaagt gagcctacca ggaatgagct gaatgaatct

    AspLysValL ysValTrpSe rArg

481 gggtagggga tctaacccag tgcctcaaag gctagcatct cccagtggag atggaatggc

541 agcagagatt gggaaagaga gagcaggaga ggccacatca gtattgttgc cgtgtatctc

601 acagaggatg ctcccacggg aagaggaagg gaccccgagg catagttcac agaccacagg

661 cagaaggttg gacttgctgg gaaaacctgg gcccaatttg ctaatttgtg aatgacttct

721 ctttcttgga ttacatttaa ctctgatgaa aaggaaagca gcatttgatc agtgaccatg

781 tgtgacggcc atctaatgca gatcgtttca ttaaatcctc ccttcattca ttccagacct

841 gcctgatagc catgttctcc ttagacaaca gatct

```

**B**

```

TAAATTAGGC CCTCTGCCCC TCTATTGGTC TAGCTCTCCA AGCTGATTAT CCGGGCTGCT

  AI047339|                      ------ ---------- ---------- ----------

  AI046376|                          -- ---------- ---------- ----------

  AA986200|                           - ---------- ---------- ----------

  AA986067|                             ---------- ---------- ----------

  AI048598|                             ---------- ---------- ----------

  AI047875|                             ---------- ---------- ----------

  AA987109|                             ---------- ---------- ----------

  AI047382|                              --------- ---------- ----------

  AA986257|                              --------- ---------- ----------

  AI046467|                              --------- ---------- ----------

  AI046373|                              --------- ---------- ----------

  AI048620|                              --------- ---------- ----------

  AI043154|                              --------- ---------- ----------

  AI046480|                               -------- ---------- ----------

```

FIGURE 2 *The 875 bp mouse Ahsg subclone delta contains* 154 *nt of 5′-flanking DNA in addition to exon* 1 *and part of the first intron*. The DNA sequence analysis shown in **Panel A** reveals a 154 bp segment upstream from the transcriptional start site, the first exon and part of the first intron. Putative transcriptional motifs [hepatic nuclear factor (HNF)-3$\beta$ and C/EBP-$\alpha$] are indicated, as well as the transcriptional start site. One of the primers used to construct the baculoviral cDNA expression clone (SSF1) is indicated. The DNA in exon 1 is indicated in uppercase. This sequence is available from GenBank, accession number AF025820. The transcriptional start site is deduced from the alignment of 14 EST clones available in the public database (**Panel B**); the most 5′ EST clone (AI047339; Sugano mouse liver EST project) is taken to define the transcriptional start site.

expressed sequence tag (EST) clones available in public databases, especially those from the Sugano mouse liver EST project (Marra *et al.*, Washington University, St. Louis, MO). In Figure 2B, we show the alignment of 14 of the more than 50 EST sequences available; the most 5′ sequence (file identifier 1450748/ud65a11.y1, accession number AI047339) is taken to define the transcriptional start site. Upstream of the cap site (+1) can be found a TATA box (ATAAATT) at the -24/−18 position, and two motifs suggestive of sites for transcription factors C/EBP-$\alpha$ (−58 to −45, CCTTTACGCAATTC) and HNF-3$\beta$ (−126 to −115, ACTTATTTGCTT). Both of these factors are abundant in liver, and associated with the expression of liver-specific genes. Alignment of the corresponding human AHSG and rat AHSG upstream sequences (Fig. 5) reveals that all of these putative transcriptional elements are conserved in the proximal upstream regions of mouse, rat, and human genes. Moreover, this alignment of the three sequences reveals that the splice donor (SD) at the 3′-end of the first exon is precisely conserved among the three species, strongly suggesting that the mouse Ahsg genomic segment in clone delta represents the true ortholog of human AHSG.

Beyond the SD site, there is little conservation of sequence between the mouse and human first intron.

The larger clone (D, 3.45 kb Bgl II insert) harbors exons 2, 3 and 4 (Figs. 3 and 4B), in addition to two elements in the intron upstream

```
   1 gatctcatat gctaacagac tgaaactatg aaaccgccaa tacttgtctg cttttctttt
          PRIMER VIV3 --->                                 |-- (CT)n ----

  61 cctgtttctg tctgtctgtc tgtctttctt cctttctttc tcttctctct ttctttctct
     ------------- microsatellite (CT)n repeat ----------------------

 121 tcctctttct ttcttcttcc ttcctttctc ttctttctct ttctttcttc cttttttcttc
     ------------- microsatellite (CT)n repeat ----------------------

 181 tttctttctc tttgctcttt ctttcccttc cttccttcct ttctcttctt tctcttctct
     ------------- microsatellite (CT)n repeat ----------------------

 241 ctttcttttc cttcctttct tccttccttc cttccttcct tccttccttc cttccttcct
     ------------- microsatellite (CT)n repeat ----------------------

 301 tccttctttc cttcttcttt ttgagacaga aaggtttctc tttgtagccc tgactgtcct
     -- (CT)n repeat -------|         |---- B1 repetitive element ----

 361 ggaacttact atgtagacca ggctggcctc aaacacacag agatccacct gcctctgcct
     Primer VIV7 -->
     ----------------------- B1 repetitive element -----------------

 421 cctggctcct ggaatcatag gcatgtgtca ccgactattt gtctgctttt aacatgcaaa
     -|

 481 gttggaaact ccatacggtt cagcttaaca taaagatgag aagaacaagt ttgtgtcact
 541 agagacttag gatttaggag gaaaataagg taaacaccag gatgctcaga gtgaggattg
 601 acaaccagct ttacaatggg acagctgatt tgaaaccacg gttttcctgg gtgagtttta
 661 agggcagttg gcaaaagacg taatggccgg ctctctgcct agtttacatg ctgaagggaa
 721 agccgtgagc gagcactgtg catgtgctac gtgctgattg tgagatgctc attatgggat
 781 gcccgagtgg atcaagaant tccctgcaca taaacccagt gcatctaccc atggttagtt
 841 ctgaggtctc cggagagtga aaatgcccag tgaactaaat tgggttgaga gttttcaaac
 901 tttggggcat ttcaaggtgt gaacggggaa tacatagaca ggtgaaacac tgaactcctc
 961 acagggtcct gcaagcttcc caaaatgctt ccatcctagt ggtgacagtt tcccagcctc
1021 agaatagaaa ggcggcaaac aggagatagg actctctgtg catccaggac ccaggaaggt
1081 agaagataaa gagccaaggg aggagcaaga gaaaccttta aggacacaaa cactcaaaga
1141 aagggagaaa agtgggcaac tagagagaag aaagaatgaa gcagaatgaa agatagcaaa
1201 gataataaac ctttcaagat aaaagctagc cctcagagtc acttctttgt aaagagagct
1261 cagaataagg agtatggctg ggacagctgt tggagcacgg cccccccctcc cgtccctttt
1321 ttccccctcc cccctcccca tttcacaccc gctccatcta tagtggaaga ctaaaaagcc
1381 aaaacaaaac aaaaagaaaa aaaaaaaaaa ccactgcagc actgcatagc tggaaggggt
1441 ggggctgaca tctccttagt cctcccaccc ctcagccaag ccccaccaca gggctagtgt
1501 tcacactagg tgccatgcat tggatcagcg gagcagggcg tttgctcacc tctcccttct
     <-- Primer VIV11

1561 gtccggctcc acagCGGCCC TTCGGAGTGG TGTATGAGAT GGAAGTTGAC ACACTGGAGA
                   SA       Primer KOAF1 -->             exon 2
                        <--- Primer VIV5 ---------
                   ArgPro PheGlyValV alTyrGluMe tGluValAsp ThrLeuGluT

1621 CCACTTGCCA TGCTTTGGAC CCCACCCCGC TGGCAAACTG TTCTGTGAGG CAGCTGACTG
     hrThrCysHi sAlaLeuAsp ProThrProL euAlaAsnCy sSerValArg GlnLeuThrG

1681 AGCACgtgag tgctgccttg tggttggttg gtgggtgggt gggtgggtgg gagctgccca
     luHis

1741 gccaccacag ttcagcaaag tgcaggtttg gctttctcca tctcccagca gccatcttgg
1801 ctagccagag agcaaagtct aaaacccgct gtgggataga tggtgccttc cccgaggttg
1861 attttcacaa cacttgggct tttcttcttg aagccctcgg gagagcagat tatgatgttt
1921 caataacacc cgtgaaggtt gccttgggca ggttacctcc cacaaccctg ccaagacgct
1981 ccctgaatga gcagccagag tatatatact gcttcagaat gccggcatct gatttcttta

2041 cccagGCGGT GGAGGGAGAC TGTGACTTCC ACATCCTGAA ACAAGACGGC CAGTTCAGGG
```

FIGURE 3 *Internal exons 2–4 of mouse Ahsg gene.* The 3.45 kb Bgl II fragment cloned pGEM4Z (clone D) was sequenced using a combination of dye primers (SP6 and T7, at the 3′ and 5′-ends of the subclone, respectively), and dye terminator reactions. Sequencing primers are indicated beneath the DNA sequence. Exons are indicated in the DNA sequence as uppercase letters. The first intron contains 271 bp of the microsatellite (C,T)*n* ∗ (A,G)*n* adjacent to a middle repetitive element of 92 bp in the B1 family. This sequence is available from GenBank, accession number AF025821.

```
                                  Primer VIV8 -------->     exon 3
     AlaVa lGluGlyAsp CysAspPheH isIleLeuLy sGlnAspGly GlnPheArgV

2101 TGATGCACAC CCAGTGTCAT TCCACCCCAG gtcagaaaac actgcctctt gtttttatct
     alMetHisTh rGlnCysHis SerThrPro

                                  SD
2161 cgtagaatga gaaaggaatc agaatagttt tgaactcaaa taggtctcac ttcctctgtg
2221 aggattctgg gtcctgggga ttctaatgcc atcttttaaa gaagcccgtt cttgtgggcg
2281 aacattgtcc ccgtggctgt gacttggtga ccttcataca gctgcttgaa ggcttagaga
2341 agaagtcatg ctacattgga gcactaggag ccctttctaa ataagcaagc tgttgtgagt

2401 acactggaga gctgcagttg agaccctgct atcttcccgc ccagACTCTG CAGAGGACGT
                                                   SA
                                                      SerA laGluAspVa

2461 TCGTAAGTTG TGCCCACGGT GCCCACTCCT GACTCCGTTC AACGATACCA ACGTGGTCCA
     lArgLysLeu CysProArgC ysProLeuLe uThrProPhe AsnAspThrA snValValHi

2521 CACCGTCAAC ACTGCCCTGG CTGCCTTCAA CACACAGAAT AATGGAACCT ATTTTAAACT
     sThrValAsn ThrAlaLeuA laAlaPheAs nThrGlnAsn AsnGlyThrT yrPheLysLe

2581 GGTGGAGATT TCCCGGGCTC AAAATGTGgt aaaaacttaa cactcttttg atagatttgg
                                  SD
     uValGluIleSerArgAlaGlnAsnValCy

2641 gcgatttggt ggcccttggg gcatgtgtgg gggtgataac cagaagaaaa ggaaacattg
2701 gctggaagaa ctggcagggg ttctagaact tatggagccc taaactctca gcagcgctgt
2761 cccaaactga gccagttcaa cataggtcag ccacaggcag aaggcaggta acaccctggc
2821 ctcctggctt tacctaacac ttaatagcag ggctctctgt tcagacacaa tacattcacc
2881 gggtgccaca cgtttacacc ctgccagtaa catctgccgc agtctgggaa tcaacactaa
2941 caaaggtatg ggcaaatact ggaaggttcc taatctgcct ttcaaatcca ggtttttgag
3001 gtgggagggg accatctaat tgtatagcca aagcaactat ttgagtgcaa tagacttgag
3061 atgtttaagg aagctggcaa tggaaataag tcaagacata cttgcaaata cattagtgta
3121 ggtggtgatt ctgtaattcc tgggacaatt cccatcccat actgcaccag gcgctgtgct
                                          <--- Primer VIV4 -------

3181 tgcaaggctc ccagcgtcag ggggaaggaa gcacagtgac ttccattttg atcctgctgt
3241 gggaaactgg ggtgggggca tcttttcact tcccgcttcg agcctgggat gactggaaca
3301 ttgaattctg aaggttgagg gccaggagat gctgggtttc catccctgcc ggactaaagt
3361 tagcctttg gcttcctgtt tcctctctga aaacttaacc tctgccccat gcgatggaca
3421 acgatctctt  gccaatactt tgagacgact  cagatc
```

FIGURE 3 (Continued).

of exon 2. The first of these elements is a 271 nt long microsatellite (T,C)$n*$(A,G)$n$ composed of 96% C or T in the coding strand. The second element found in this intron is a 92 bp sequence with high homology to the family of B1 middle repetitive elements characterisitic of mouse DNA.[41,42]

### Chromosomal Mapping of Mouse Ahsg

In order to map the mouse Ahsg gene to its chromosomal site, we targetted a PCR amplicon of 198 bp located in the 3′-UTR of the mouse Ahsg cDNA; using a site in the 3′-UTR was used to increase the possibility of finding sequence polymorphism between different mouse strains.[43,44] The data indicate a localization of the Ahsg gene to chromosome 16 at approximately 16 cM, adjacent to Dagk3 (Diacylglycerol kinase 3) gene, a position syntenic with human chromosome 3q27, the location of the human AHSG gene.[21,45] The mapping profile of the Ahsg gene, with respect to the genes already mapped on chromosome 16, and a composite map of the Jackson BSS panel Map, *versus* the MGD composite map are depicted in (Fig. 6).

### Expression of Mouse Ahsg cDNA as a Recombinant Baculovirus and Assay for Insulin Receptor Tyrosine Kinase Activity

A baculoviral transfer vector, pMusBac$\alpha$3 (11,440 bp) was created using a 1.2 kb BamHI-XbaI segment of the mouse Ahsg cDNA featuring an intact 1035 bp open reading frame, as described in Materials and Methods. This transfer vector (2 μg) was transfected into Sf 9 cells along with 5 μg of the wild-type baculoviral

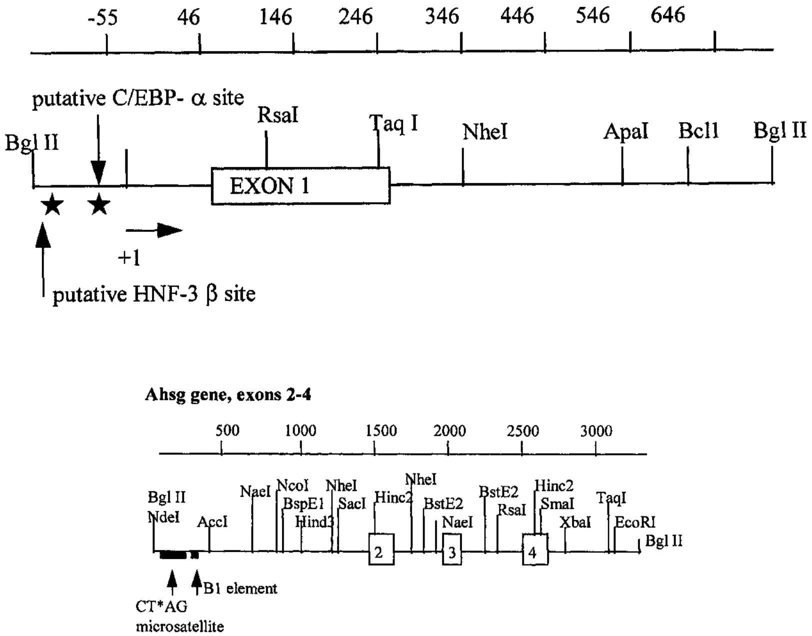

FIGURE 4 *Schematic structure of subclones delta and D.* Shown in **Panel A** is the schematic structure of the upstream subclone delta whose sequence is shown in Figure 2A. Putative transcriptional motifs (hepatic nuclear factor (HNF)-3$\beta$ and C/EBP-$\alpha$) are indicated, as well as the transcriptional start site. The segment of the first exon which encodes the first 85 amino acids of mouse $\alpha_2$-HS-glycoprotein is shown in the shaded box. Shown in **Panel B** is the schematic structure of exons 2, 3 and 4, including part of the first intron and part of the intron downstream from exon 4. Shaded boxes indicate the exons. The 271 bp microsatellite (C,T)*n* * (A,G)*n* and the 92 bp middle repetitive (*B*1 family) element are indicated by bracketing.

DNA, and blue plaques selected from 2% agarose plugs stained in Bluo-Gal. Twice-purified viral plaques were expanded at 27°C, and recombinant viral stocks checked for homogeneity using PCR (baculoviral primers −44, 5′-TTTACTGTTTTCGTAACAGTTTTG-3′OH; and +778, 5′-CAACAACGCACAGAATCTAGC-3′OH). Expanded recombinant baculoviral stocks were used to transfect cabbage looper HighFive™ cells, and supernatants collected after 48–72 h. Supernatants were purified using affinity chromatography on jacalin lectin columns and proteins eluted with 25 μM melibiose were separated on 12% SDS-PAGE and electroblotted to nitrocellulose. Probing of this membrane with a polyclonal rabbit antibody specific for rat fetuin reveals two prominent bands of 60 and 66 kD (Fig. 7A) in addition to a fainter band of a slightly smaller apparent molecular weight.

### Inhibition of Insulin Receptor Tyrosine Kinase Activity

Jacalin lectin affinity-purified recombinant mouse $\alpha_2$-HSG (Fig. 7A) was used to test inhibitory activity of the insulin receptor in a TK assay. Mouse $\alpha_2$-HSG was tested at concentrations ranging from 5 μg/ml to 20 μg/ml in the presence of insulin. Approximately 70% inhibition of IR-TK activity was observed at 15 μg/ml (Fig. 7B).

### Inhibition of Insulin-stimulated IR Autophosphorylation and Insulin-stimulated DNA Synthesis

Multiple experiments were performed in duplicate in order to verify the ability of recombinant mouse $\alpha_2$-HSG to inhibit autophosphorylation of the 95 kD $\beta$-subunit of the IR. The proteins were

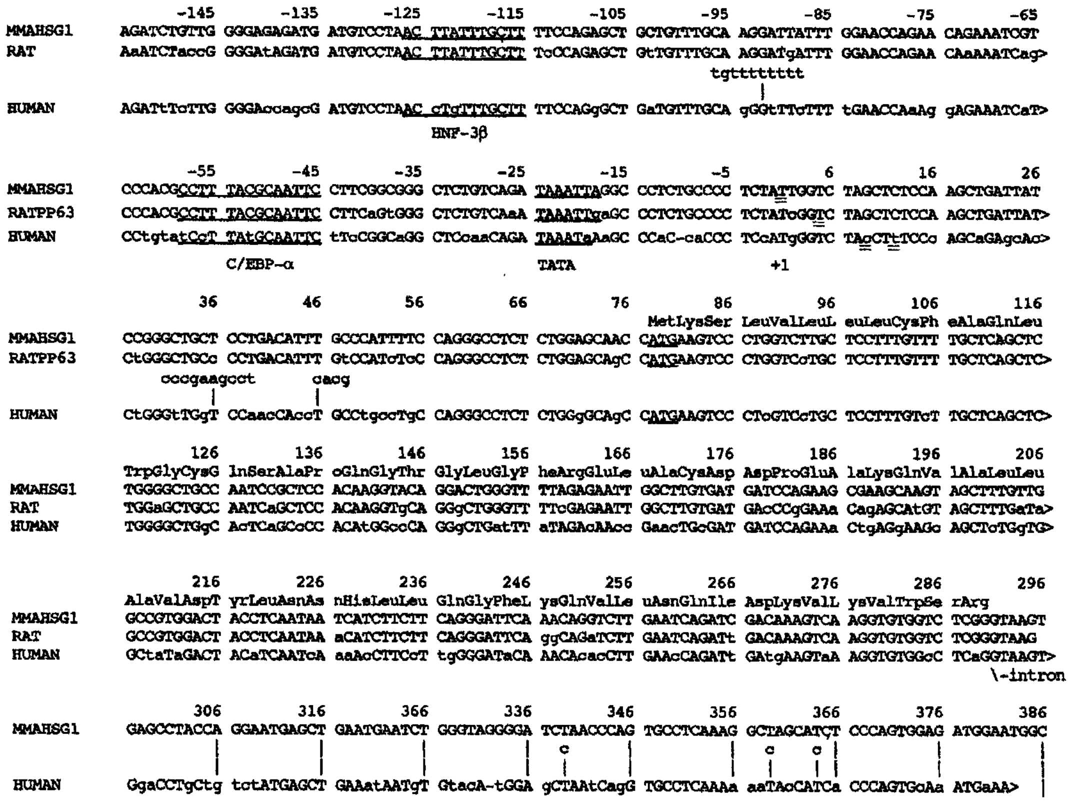

FIGURE 5 *Alignment of the proximal promoter sequences of mouse, rat, and human AHSG genes reveals an evolutionary conservation of putative transcription factor motifs, TATA box, and splice donor (SD) sequences.* The mouse sequence is taken from Figure 2A; the rat sequence is taken from GenBank files M36547 and X63446;[49,26] the human sequence is taken from GenBank files Y09540 and M16961.[55,21] Sequences were aligned using CLUSTAL. Transcriptional start site are indicated by double underlining. The C/EBP-$\alpha$ and HNF-3$\beta$ motifs and the MET initiator codon are indicated by single underlining. The conceptual translation of the mouse Ahsg sequence is indicated above the line MMAHSG1. Where the rat or human sequence agrees with the nucleotide in the mouse sequence, it is capitalized; otherwise, mismatched bases are indicated in lowercase in the human and rat sequence lines. The human sequence has two insertions relative to the mouse sequence; the rat sequence has a single deletion relative to the mouse sequence, filling in the gap with a (−). The numbering above the mouse sequence line is relative to the mouse transcriptional start site defined in Figure 2.

A

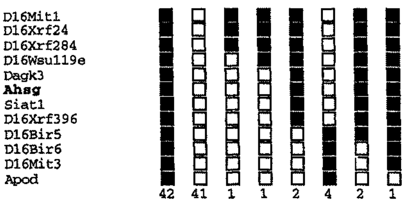

FIGURE 6 *Chromosomal mapping of the mouse Ahsg gene obtained from typing patterns derived from PCR analysis using the Jackson Laboratories BSS interspecific backcross.* Locations of allele types of C57B1/6J or *M. spretus* were determined by the composite map of backcross panels. Mouse Ahsg is mapped to chromosome 16 at 16 centimorgans. Mapping profile of the Ahsg gene with respect to other genes mapped to chromosome 16 (**Panel A**). Demonstration of a composite map of the Jackson BSS panel Map *versus* the MGD composite map. The Ahsg gene is localized at approximately 16 cM closely to the Dagk3 gene (**Panel B**).

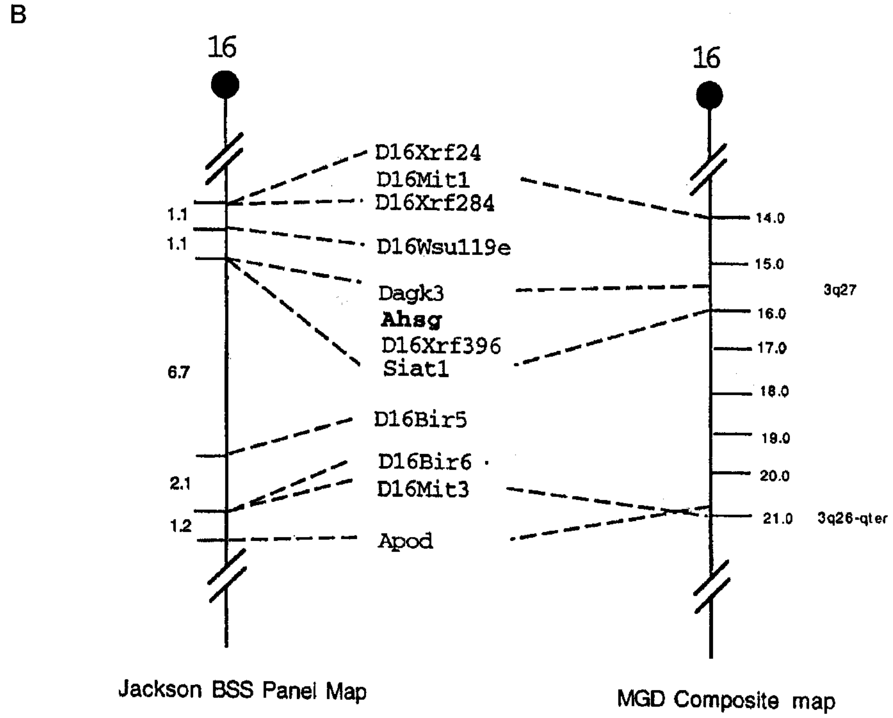

FIGURE 6 (Continued).

separated on a 10% SDS-PAGE and the $^{32}P$ incorporated was detected by autoradiography of the dried gel. Mouse $\alpha_2$-HSG at a concentration of 1 μg/ml completely abolished insulin-induced autophosphorylation of the $\beta$-subunit of partially purified rat IR (Fig. 7C). Various concentrations of recombinant mouse $\alpha_2$-HSG were tested for their ability to inhibit

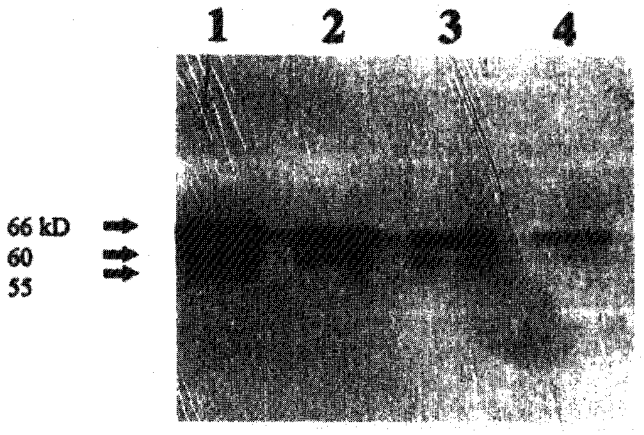

FIGURE 7A *Recombinant mouse* $\alpha_2$-HS-glycoprotein expressed in a baculoviral system. Immunoblot of mouse $\alpha_2$-HSG synthesized in insect cells, partially purified by lectin chromatography. Numbers below the lanes indicate fractions eluted from the jacalin affinity column. Two forms are prominent, with apparent molecular weights of 60–66 kD.

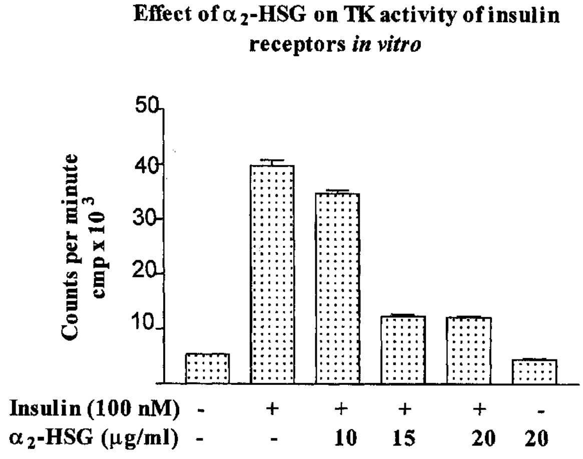

FIGURE 7B *Recombinant mouse* $\alpha_2$-HSG is an inhibitor of the insulin receptor (IR) at the tyrosine kinase (TK) level. The bar graph indicates that the tyrosine kinase activity of the insulin receptor is inhibited by $\alpha_2$-HSG. IRs purified from H-35 rat hepatoma cells were incubated for 30 min, 20°C with various concentrations of mouse $\alpha_2$-HSG (10, 15 or 20 μg/ml) in the presence or absence of insulin (100 nM). Insulin-stimulated IR-TKA was assessed by its ability to transfer $^{32}P$ onto the synthetic substrate poly ($Glu^{80}Tyr^{20}$). Lane 1 represents stimulation of the insulin receptor with 100 nM insulin. Mouse $\alpha_2$-HSG inhibits over 70% the insulin-stimulated receptors at a concentration of 15 μg/ml. The results shown are representative of three separate experiments.

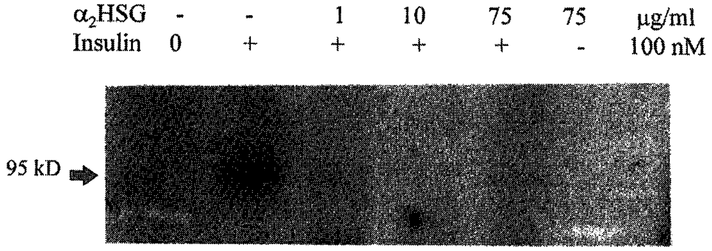

FIGURE 7C *Effect of* $\alpha_2$-HSG on autophosphorylation of the IR *in vitro*. The autoradiograph reveals that recombinant mouse $\alpha_2$-HSG inhibits the autophosphorylation of the insulin receptor. Partially purified insulin receptors from H-35 cells were pre-incubated with or without insulin (100 nM) in presence or absence of mouse $\alpha_2$-HSG (1, 10, 75 μg/ml) respectively. Autophosphorylation was determined as described in Materials and Methods. The position of the 95 kDa $\beta$-subunit of the IR is indicated. Insulin-induced autophosphorylation of the $\beta$-subunit of the IR compared to basal (lanes 2 and 1, respectively). Purified mouse protein at concentrations of 1 μg/ml completely abolished insulin-induced autophosphorylation of the IR.

insulin-induced DNA synthesis. Mouse $\alpha_2$-HSG at 25–35 μg/ml completely abolished insulin-induced DNA synthesis in H-35 hepatoma cells (Fig. 8).

### DISCUSSION

The human glycoprotein $\alpha_2$-HSG acts as a specific inhibitor of the human IR, at the level of the TK.[18, 46, 47] In this study, we have cloned the mouse homolog of human AHSG, determined its genomic organization, chromosomal location and demonstrated its inhibitory activity towards the IR-TK.

The mouse clone we have isolated by screening a mouse Svj/129 genomic library (λKOA-1B), harbors 18.6–23.0 kb of the mouse Ahsg gene. Sequence analysis shows that the mouse gene is organized with the same exon–intron organization as both the rat fetuin gene[48, 49] and the human AHSG gene.[50] Both the rat

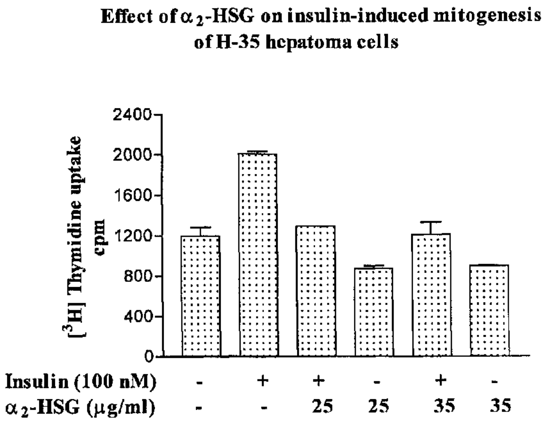

FIGURE 8 *Mouse* $\alpha_2$-HSG inhibits insulin-induced mitogenesis on H-35 hepatoma cells. The data describe the effect of recombinant mouse $\alpha_2$-HSG on insulin-induced mitogenesis of H-35 rat hepatoma cells. Subconfluent dishes were treated as described in Materials and Methods and the uptake of [$^3$H]-thymidine was measured. Insulin induced DNA synthesis (represented in the first bar). Insulin-stimulated mitogenesis is completely blocked at concentrations of 25–35 μg/ml of mouse $\alpha_2$-HSG (bar 3 and 5). The $p$ value was calculated by comparing the second bar, insulin-stimulated mitogenesis and the third bar, $\alpha_2$-HSG (25 μg/ml) and insulin-treated cells (the $P < 0.05$, $p = 0.008$ is considered significant). A comparison between the second bar, insulin-stimulated cells and the fifth bar, $\alpha_2$-HSG (35 μg/ml) and insulin-treated cells demonstrated $P < 0.01$, $p = 0.008$.

fetuin gene and the human AHSG gene feature 7 exons. This genomic subclones we have sequenced cover exon 1 (Figs. 2A and 4A) and exons 2–4 (Figs. 3 and 4B). We have not sequenced clone λKOA-1B downstream of exon 4, and we are unable to address the question of exon–intron organization downstream of exon 4. In all three species, intron 1 occurs at the same codon position and features the same splice acceptor (SA). Our sequence data demonstrate that intron 1 is 2000 bp in length in the mouse, slightly larger than the 1.7 kb reported for the corresponding intron in the rat,[49] and slightly smaller that the corresponding intron in the human gene (2.3 kb;[50]). Clone λKOA-1B also contains a 271 bp tract of $(T,C)n * (A,G)n$ adjacent to a B1 element of 92 bp. Numerous copies of B1, B2 and D1 elements are found in rodent genomes.[41,42] Some of these B1 elements are not neutral relics of evolutionary spread of the DNA element, but may play a functional role in the regulation of the genes in which they are present. For example, B1 elements can act as negative regulators of gene expression.[51,52] Moreover, androgen regulation of one gene seems to have been acquired during evolution through the insertion of a B1 repetitive element into the transcription unit.[53] Whether the B1 element in the first mouse Ahsg intron plays a role in the regulation of Ahsg transcription has not yet been addressed experimentally. The conservation of C/EBP-$\alpha$ and HNF-3$\beta$ binding sites in the proximal 5′ upstream region of the mouse and human AHSG gene is likely to have a functional significance. Falquerho *et al.*[54] have shown these motifs to be functional in transient transfection of the rat AHSG gene. Moreover, Banine *et al.*[55] have analyzed the human AHSG promoter–enhancer in transient transfection assays as well, suggestive of a functional role for the C/EBP-$\alpha$ and HNF-3$\beta$ sites in the human gene. The putative HNF-3$\beta$ site in the mouse Ahsg gene is conserved between mouse, rat and human. Given the role of human,[18] rat,[48] and mouse (this study) proteins in the inhibition of insulin receptor function, it is intriguing to note that one form of maturity onset diabetes of the young (MODY), maps to the gene encoding HNF-3$\beta$ in man.[56–58] Another site conserved between the rat, mouse, and human genes is the putative C/EBP-$\alpha$ site at −58 to −45 (CCTTTACGCAATTCC in mouse). This site has been implicated in the cytokine-induced down regulation of the rat fetuin gene associated with the acute phase.[59]

We have successfully employed the 3′ UTR of the mouse Ahsg cDNA as a target for interspecies polymorphism. The 3′ UTR region was chosen because it is not disrupted by introns in the human gene,[50] and, therefore, the primer pairs designed from the 3′ short end of the cDNA should amplify the same 198 bp fragment from the genomic DNA. The 3′ UTR sequences constitute a rich source of genetic markers for the mouse genome.[43] Using this PCR based mapping technique, and panels between *C57BL/6J* and *Mus spretus*, the mouse Ahsg gene was localized to chromosome 16 at 16 cM. This location is syntenic with the position of the human AHSG gene on chromosome 3 band q27. It is interesting to note that the mouse Ahsg maps in the vicinity of other genes implicated in signal transduction and gene regulation such as Dagk3 (diacylglycerol kinase 3, gamma 3, 15.5 cM), Prkml (protein kinase, mitogen activated kinase 1, 14.5 cM), and EIF4$\beta$, eukaryotic translation initiation factor 4B, 14.2 cM). These genes have also been mapped at the syntenic position in man.

In the present study, we have used the baculoviral system to express mouse $\alpha_2$-HSG as a recombinant protein. The baculoviral protein has an apparent molecular weight of 60–66 kD and has been shown to block insulin-induced IR-autophosphorylation at 1 μg/ml, IR-TKA at 15 μg/ml and insulin stimulated mitogenesis at 25 μg/ml *in vivo*. In the rat, it has been shown that pp63/fetuin can inhibit IR-TK and IR autophosphorylation with a half-maximal inhibition of 0.24 μg/ml.[48] These results demonstrate that the IR inhibitory activity of rat fetuin,[48] human $\alpha_2$-HSG,[18, 46] and bovine fetuin[60] now extends to mouse $\alpha_2$-HSG. Together, these results suggest that the mouse Ahsg gene is the ortholog of the human AHSG gene. Additional studies will be necessary to determine whether the *in vitro* demonstration of IR inhibitory activity demonstrated now for four of these proteins (rat, bovine, human and mouse) has a physiological significance for glucose regulation in these species.

### *Acknowledgements*

This work was supported by an NIH R01 awarded to G.G. (DK44382), an NIH predoctoral fellowship (F31 DK0952) awarded to V.J.C., and a Blue Cross Blue Shield Foundation Award Grant (#241-SAP/97) awarded to V.J.C. The authors wish to thank Dr. Robert Thomas, and Ms. Kassandra McGehee at the Center for Molecular Medicine and Genetics for the excellent technical support in the isolation of the mouse Ahsg clone and Dr. Suresh Mathews and Dr. Myron A. Leon for the excellent assistance in the manuscript preparation. Also, we thank Dr. Roger Askew at the University of Cincinnati for the kind gift of the mouse genomic library and Dr. Michael Hagen (Center for Molecular Medicine and Genetics) for DNA sequencing.

### *References*

[1] Heremans, J. F. (1960). *Les Globulines Seriques du Système Gamma*, pp. 103–107. Arcia, Brussels.
[2] Schmid, K. and Burgi, W. (1961). Preparation and proliferation of the human plasma Ba-$\alpha_2$-HS glycoprotein, *Biochim. Biophys. Acta*, **47**, 440–453.
[3] Lewis, J. G., *Ph.D. Thesis* (1983). pp. 1–1885, University of Otago, Dunedin, New Zealand.
[4] Lebreton, J. P., Joisel, F., Raoult, J. P., Lannuzel, B., Rogez, J. P. and Humbert, G. (1979). Serum concentration of human $\alpha_2$-HS-glycoprotein during the inflammatory process, *J. Clin. Invest.*, **64**, 1118–1129.
[5] Ashton, B. A. and Smith, R. (1980). Plasma $\alpha_2$-HS-glycoprotein concentration in Paget's disease of bone: its possible significance, *Clin. Sci.*, **58**, 435–438.
[6] Kalabay, L., Cseh, K., Benedek, S., Fakete, S., Masszi, T., Herjeczki, K., Pozsonyi, T., Jakab, L. and Kakab, L. (1991). Serum alpha$_2$-HS glycoprotein concentration in patients with hematological malignancies, *Ann. Hematol.*, **63**, 264–269.
[7] Van Oss, C. J., Gillman, C. F., Bronson, P. M. and Border, J. R. (1974). Opsonic properties of human serum alpha$_2$ HS-glycoprotein, *Immunol. Commun.*, **3**, 329–335.
[8] Quelch, K. J., Cole, W. G. and Melick, R. A. (1984). Noncollagenous proteins in normal and pathological human bone, *Calcif. Tissue. Int.*, **36**, 545–549.
[9] Malone, J. D., Teitelbaum, S. L., Griffin, G. L., Senior, R. M. and Kahn, A. J. (1982). Recruitment of osteoclast presursors by purified bone matrix constituents, *J. Cell Biol.*, **92**, 227–230.
[10] Lewis, J. G. and Andre, C. M. (1980). Effect of human $\alpha_2$-HS-glycoprotein on mouse macrophage function, *Immunology*, **39**, 317–322.
[11] Lewis, J. G. and Andre, C. M. (1981). Enhancement of human monocyte phagocytic function by $\alpha_2$-HS-glycoprotein, *Immunology*, **42**, 481–487.
[12] Dickson, I. R., Poole, A. R. and Veis, A. (1975). Localization of plasma $\alpha_2$-HS-glycoprotein in mineralising human bone, *Nature*, **256**, 430–432.
[13] Schinke, T., Amendt, C., Trindl, A., Pöschke, O., Müller-Esterl, W. and Jahnen-Dechent, W. (1996). The serum protein $\alpha_2$-HS glycoprotein/fetuin inhibits apatite formation *in vitro* and in mineralizing calvaria cells, *J. Biol. Chem.*, **271**, 20789–20796.

[14] Triffitt, J. T., Gebauer, U., Ashton, B. A., Owen, M. E. and Reynolds, J. J. (1976). Origin of plasma $\alpha_2$-HS-glycoprotein and its accumulation in bone, *Nature*, **262**, 226–227.

[15] Mbuyi, J. M., Dequeker, J., Bloemmen, F. and Stevens, E. (1982). Plasma proteins in human cortical bone: enrichment of $\alpha_2$-HS-glycoprotein, $\alpha_1$ acid-glycoprotein, and IgE, *Calcif. Tissue. Int.*, **34**, 229–231.

[16] Ashton, B. A., Höhling, H. J. and Triffitt, J. T. (1976). Plasma proteins present in human cortical bone: enrichment of the $\alpha_2$-HS-glycoprotein, *Calcif. Tissue. Res.*, **22**, 27–33.

[17] Keely, F. and Sitarz, E. E. (1985). Characterization of proteins from the calcified matrix of atherosclerotic human aorta, *Atherosclerosis*, **46**, 29–40.

[18] Srinivas, P. R., Wagner, A. S., Reddy, L. V., Deutsch, D. D., Leon, M. A., Goustin, A. S. and Grunberger, G. (1993). Serum $\alpha_2$-HS-glycoprotein is an inhibitor of the human insulin receptor at the tyrosine kinase level, *Mol. Endo.*, **7**, 1445–1455.

[19] Jahnen-Dechent, W., Schinke, T., Trindl, A., Müller-Esterl, W., Sablitzky, F., Kaiser, S. and Blessing, M. (1997). Cloning and targeted deletion of the mouse fetuin gene, *J. Biol. Chem.*, **272**(50), 31496–31503.

[20] Dziegielewska, K. M., Brown, W. N., Casey, S. J., Christie, D. L., Foreman, R. C., Hill, R. M. and Saunders, N. R. (1990). The complete cDNA and amino acid sequence of bovine fetuin. Its homology with $\alpha_2$-HS glycoprotein and relation to other members of the cystatin superfamily, *J. Biol. Chem.*, **265**, 4354–4357.

[21] Lee, C. C., Bowman, B. H. and Yang, F. (1987). Human $\alpha_2$-HS-glycoprotein: The A and B chains with connecting sequence are encoded by a single mRNA transcript, *PNAS USA*, **84**, 4403–4407.

[22] Kellerman, J., Haupt, H., Auerswald, E. A. and Müller-Ester, W. (1989). The arrangement of disulfide loops in human $\alpha_2$-HS-glycoprotein. Similarity to the disulfide bridge structures of cystatins and kininogens, *J. Biol. Chem.*, **264**, 14121–14128.

[23] Elzanowski, A., Barker, W. C., Hunt, L. T. and Seibel-Ross, E. (1988). Cystatin domains in $\alpha_2$-HS-glycoprotein and fetuin, *FEBS Letters*, **227**, 167–170.

[24] Yoshioda, Y., Gejyo, F., Marti, T., Rickli, E. E., Bürgi, W., Offner, G. D., Troxler, R. F. and Schmid, K. (1986). The complete amino acid sequence of the A-chain of human plasma $\alpha_2$-HS-glycoprotein, *J. Biol. Chem.*, **261**, 1665–1676.

[25] Dziegielewska, K. M., Mollgard, K., Reynolds, M. L. and Saunders, N. R. (1987). A fetuin-related glycoprotein ($\alpha_2$HS) in human embryonic and fetal development, *Cell Tissue. Res.*, **248**(1), 33–41.

[26] Rauth, G., Pöschke, O., Fink, E., Eulitz, M., Tippmer, S., Kellerer, M., Haring, H. U., Nawratil, P., Haasemann, M., Jahnen-Dechent, M. and Müller-Esterl , W. (1992). The nucleotide and partial amino acid sequences of rat fetuin: identity with the natural tyrosine kinase inhibitor of the rat insulin receptor, *Eur. J. Biochem.*, **204**, 523–529.

[27] Brown, W. M., Dziegielewska, K. M., Saunders, N. R., Christie, D. L., Nawratil, P. and Müller-Esterl, W. (1992). The nucleotide and deduced amino acid structures of sheep and pig fetuin: common structural features of the mammalian fetuin family, *Eur. J. Biochem.*, **205**, 321–331.

[28] Christie, D. L., Dziegielewska, K. M., Hill, R. M. and Saunders, N. R. (1987). Fetuin: the bovine homologue of human $\alpha_2$-HS-glycoprotein, *FEBS Letters*, **214**, 45–49.

[29] Hayase, T., Rice, K. G., Dziegielewska, K. M., Kuhlenschmidt, M., Reilly, T. and Lee, Y. C. (1992). Comparison of *N*-glycosides of fetuins from different species and human $\alpha_2$-HS-glycoprotein, *Biochemistry*, **31**, 4915–4921.

[30] Le Cam, A., Magnaldo, I., Le Cam, G. and Auberger, P. (1985). Secretion of major phosphorylated glycoprotein by hepatocytes. Characterization of specific antibodies and investigation of the processing, excretion kinetics, and phosphorylation, *J. Biol. Chem.*, **260**, 15965–15971.

[31] Le Cam, A., Auberger, P., Falquerho, L., Contreres, J. O., Pages, G., Le Cam, G. and Ross, B. (1992). pp63 is very likely the rat fetuin, *Cell*, **68**, 8.

[32] Le Cam, A. and Le Cam , G. (1989). A new negatively regulated acute phase phosphoprotein synthesized by rat hepatocytes, *Biochem. J.*, **230**, 603–607.

[33] Haasemann, M., Nawratil, P. and Müller-Esterl, W. (1991). Rat tyrosine kinase inhibitor shows sequence similarity to human $\alpha_2$-HS-glycoprotein and bovine fetuin, *Biochem. J.*, **274**, 899–902.

[34] Yang, F., Chen, Z. L., Bergeron, J. M., Cupples, R. L. and Friedrichs, W. E. (1992). Human $\alpha_2$-HS-glycoprotein/bovine fetuin homologue in mice: identification and developmental regulation of the gene, *Biochim. Biophys. Acta*, **1130**, 149–156.

[35] Terkelsen, O. B. F., Jahnen-Dechent, W., Nielsen, H., Moos, T., Fink, E., Nawratil, P., Müller-Esterl, W. and Mollgard, K. (1998). Rat fetuin: distribution of protein and messenger RNA in embryonic and neonatal rat tissues, *Anatomy and Embryology*, **197**(2), 125–133.

[36] Lowe, T., Sharefkin, J., Yang, S. Q. and Dieffenbach, C. W. (1990). A computer program for selection of oligonucleotides primers for polymerase chain reactions, *Nucleic Acids Res.*, **18**, 1757–1761.

[37] Ko, M. S. H., Wang, X., Horton, J. H., Hagen, M. D., Takahashi, N., Maezaki, Y. and Nadeau, J. H. (1994). Genetic mapping of 40 cDNA clones on the mouse genome by PCR, *Mamm. Genome*, **5**, 349–355.

[38] Rowe, L. B., Nadeau, J. H., Turner, R., Frankel, W. N., Letts, V. A., Eppig, J. T., Ko, M. S. H., Thurston, S. J. and Birkenmeier, E. H. (1994). Maps from two interspecific backcross DNA panels available as a community genetic mapping resource, *Mamm. Genome*, **5**, 253–274.

[39] Manly, K. F. (1993). A Macintosh program for storage and analysis of experimental genetic mapping data, *Mamm. Genome*, **4**, 303–313.

[40] Freidenberg, G. R., Klein, H. H., Cordera, R. and Olefsky, J. M. (1985). Insulin receptor kinase activity in rat liver, *J. Biol. Chem.*, **260**, 12444–12453.

[41] Quentin, Y. (1989). Successive waves of fixation of B1 variants in rodent lineage history, *J. Mol. Evol.*, **28**, 299–305.

[42] Labuda, D., Sinnett, D., Richer, C., Deragon, J. M. and Striker, G. (1991). Evolution of mouse B1 repeats: 7SL RNA folding pattern conserved, *J. Mol. Evol.*, **32**, 405–414.

[43] Takahashi, N. and Ko, M. S. H. (1993). The short 3′-end region of complementary DNAs as PCR-based polymorphic markers for an expression map of the mouse genome, *Genomics*, **16**, 161–168.

[44] Levitt, R. C. (1991). Polymorphisms in the transcribed 3′ untranslated region of eukaryotic genes, *Genomics*, **11**, 484–489.

[45] Rizzu, P. and Baldini, A. (1995). Three members of human cystatin gene superfamily, AHSG, HRG, and KNG, map within one megabase of genomic DNA at 3q27, *Cytogent. Cell Genet.*, **70**, 26–28.

[46] Srinivas, P. R., Goustin, A. S. and Grunberger, G. (1995). Baculoviral expression of a natural inhibitor of the human insulin receptor tyrosine kinase, *Biochem. Biophys. Res. Commun.*, **208**, 879–885.

[47] Srinivas, P. R., Deutsch, D. D., Mathews, S. T., Goustin, A. S., Leon, M. A. and Grunberger, G. (1996). Recombinant human $\alpha_2$-HS glycoprotein inhibits insulin-stimulated mitogenic pathway without affecting metabolic signaling in Chinese Hamster Ovary cells overexpressing the human insulin receptor, *Cell. Signal.*, **8**, 567–573.

[48] Auberger, P., Falquerho, L., Contreres, J. O., Pages, G., Le Cam, G., Rosii, B. and Le Cam, A. (1989). Characterization of a natural inhibitor of the insulin receptor tyrosine kinase: cDNA cloning, purification, and anti-mitogenic activity, *Cell*, **58**, 631–640.

[49] Falquerho, L., Patey, G., Paquereau, L., Rossi, V., Lahuna, O., Szpirer, J., Szpirer, C., Lavan, G. and Le Cam, A. (1991). Primary structure of the rat gene encoding an inhibitor of the insulin receptor tyrosine kinase, *Gene*, **98**, 209–216.

[50] Osawa, M., Umetsu, K., Sato, M., Ohki, T., Yukawa, N., Suzuki, T. and Takeichi, S. (1997). Structure of the gene encoding human $\alpha_2$-HS glycoprotein (AHSG), *Gene*, **196**, 121–125.

[51] Saksela, K. and Baltimore, D. (1993). Negative regulation of immunoglobin kappa light chain gene transcription by a short sequence homologous to the murine B1 repetitive element, *Mol. Cell Biol.*, **193**, 3698–3705.

[52] Winoto, A. and Baltimore, D. (1989). Lineage-specific expression of the T cell receptor gene by nearby silencers, *Cell*, **59**, 649–655.

[53] King, D., Snider, L. D. and Lingrel, J. B. (1986). Polymorphism in an androgen-regulated mouse gene is the result of the insertion of a B1 repetitive element into the transcription unit, *Mol. Cell Biol.*, **6**, 209–217.

[54] Falquerho, L., Paquereau, L., Vilarem, M. J., Galas, S., Patey, G. and Le Cam, A. (1992). Functional characterization of the promoter of pp63, a gene encoding a natural inhibitor of the insulin receptor tyrosine kinase, *Nucleic Acids Res.*, **20**(8), 1983–1990.

[55] Banine, F., Gangneux, C., Lebreton, J. P., Frebourg, T. and Salier, J. P. (1998). Structural and functional analysis of the 5′-transcription control region for the human $\alpha_2$-HS glycoprotein gene, *Biochim. Biophys. Acta*, **1398**(1), 1–8.

[56] Hani, E. H., Suaud, L., Boutin, P., Chevre, J. C., Durand, E., Philippi, A., Demenais, F., Vionnet, N., Furuta, H., Velho, G., Bell, G. I., Laine, B. and Froguel, P. (1998). A missense mutation in hepatocyte nuclear factor-4 alpha, resulting in a reduced transactivation activity, in human late-onset non-insulin-dependent diabetes mellitus, *J. Clin. Invest.*, **101**, 521–526.

[57] Lindner, T., Gragnoli, C., Furuta, H., Cockburn, B. N., Petzold, C., Rietzsch, H., Weiss, U., Schulze, J. and Bell, G. I. (1997). Hepatic function in a family with a nonsense mutation (R154X) in the hepatocyte nuclear factor-4 alpha/MODY1 gene, *J. Clin. Invest.*, **100**, 1400–1405.

[58] Kaisaki, P. J., Menzel, S., Lindner, T., Oda, N., Rjasanowski, I., Sahm, J., Meincke, G., Schultze, J., Schmechel, H., Petzold, C., Ledermann, H. M., Sachse, G., Boriraj, V. V., Menzel, R., Kerner, W., Turner, R. C., Yamagata, C. and Bell, G. I. (1997). Mutations in the hepatocyte nuclear factor-1$\alpha$ gene in MODY and early-onset NIDDM: evidence for a mutational hotspot in exon 4, *Diabetes*, **46**, 528–535.

[59] Akhoundi, C., Amiot, M., Auberger, P., Le Cam, A. and Rossi, B. (1994). Insulin and interleukin differentially regulate pp63, an acute phase phosphoprotein in hepatoma cell line, *J. Biol. Chem.*, **269**, 15925–15930.

[60] Mathews, S. T., Srinivas, P. R., Leon, M. A. and Grunberger, G. (1997). Bovine fetuin is an inhibitor of insulin receptor tyrosine kinase, *Life Sci.*, **61**, 1583–1592.

